# The effect of the Ankle Pump Exercise (APE) counter system assisted ankle pump motion in patients after femoral neck fracture

**DOI:** 10.1186/s12891-023-06869-x

**Published:** 2023-11-30

**Authors:** Jiaping Shi, Xia Weng, Caixia Liu, Yanzhi Ge, Lan Chai, Xuanliang Ru, Yingxing Yue, Xiaoqin Huang

**Affiliations:** 1 Department of Orthopedics, Zhejiang Hospital, 1229 Gudun Road, Hangzhou, 310030 China; 2 Department of Urology, Zhejiang Hospital, Hangzhou, 310030 China; 3 Department of Nursing, Zhejiang Hospital, Hangzhou, 310030 China; 4 Department of Rheumatology and Immunology, Zhejiang Hospital, Hangzhou, 310030 China; 5 Zhejiang Provincial Key Lab of Geriatrics, Department of Geriatrics, Zhejiang Hospital, Hangzhou, 310030 China

**Keywords:** Ankle pump exercise, Compliance, Self-management efficacy, Venous thromboembolism

## Abstract

**Objective:**

To explore the effect of the Ankle Pump Exercise (APE) counter system on moderate to high-risk Venous thromboembolism (VTE) after femoral neck fracture surgery.

**Methods:**

From June 2021 to June 2022, a total of 140 patients with moderate and high-risk VTE after femoral neck fracture surgery treated at the Department of Orthopedics of a tertiary hospital in Zhejiang were included and divided into observation (70 cases) and control (70 cases) groups according to whether APE counter system was used or not. The control group was given routine oral propaganda, and the observation group was given a comprehensive nursing intervention with APE counter system on the basis of the control group’s treatment. The compliance rates of the two groups on the postoperative 3st, 5rd, and 7th days were compared. Moreover, the General self-efficacy scale (GSES) was used to evaluate self-efficacy before and after exercise.

**Results:**

The compliance rates of the control group and the observation group on the postoperative 3st, 5rd, and 7th days were 74.3% vs. 85.7%, 67.1% vs. 85.7%, and 61.4% vs. 82.9%. On the 5rd and 7th days, the compliance of the observation group was obviously higher than that of the control group. Moreover, the mean postoperative GSES score was also significantly higher than that in the control group (23.20 ± 3.516 vs. 25.31 ± 4.583, P < 0.05, values are expressed in mean ± standard).

**Conclusion:**

APE counter system can significantly improve the compliance and self-efficacy of patients with moderate and high-risk VTE after lower limb fracture surgery.

## Introduction

Venous thromboembolism (VTE), including deep vein thrombosis (DVT) and pulmonary embolism (PE), is a multifactorial disease with characteristic clinical manifestations, phenotypic heterogeneity, mixed risk factors, difficult treatment decision-making, and high incidence of complications. The annual incidence of VTE is approximately 1‰ every year [1–3]. It is also a commonly reported complication after major orthopedic surgeries and an important factor of patients’ perioperative and unexpected death in the hospital [4]. Without early intervention, VTE can seriously affect patients’ postoperative rehabilitation and increase the associated financial burden of patients. Moreover, mortality and quality of life are significantly related to the development of PE and post-thrombotic syndrome (PTS) [5–7].

Basic prevention, physical prevention, and medication prevention have been acknowledged as the key preventative methods for DVT, according to the current guidelines for DVT prevention in orthopedics major procedures [8]. Compared to another two kinds of preventions, basic prevention is convenient, effective, economical, no time and place restrictions, simple and easy to operate and so on, especially for patients with medium and high risk of DVT. Due to its simplicity, ease of use, and affordability, ankle pump exercise (APE) has traditionally been regarded as the most common and simple mechanical preventative approach [9–11]. It remarkably accelerates the return velocity of the venous blood and increases the quantity of blood reflux [12]. Through ankle joint movement, APE makes the calf muscles contract and relax rhythmically, squeezing the venous plexus to promote the venous blood return in the lower limbs [13, 14].

APE in the early postoperative period can accelerate the blood flow in the hypercoagulable state and avoid blood stasis [15, 16]. Moreover, the incidence of lower limb DVT was significantly lower than that of the control group, indicating that APE can significantly enhance the postoperative blood hypercoagulable state [17].

The incidence of VTE is high after hip fracture surgery especially among elderly patients, with an estimated incidence of 46-60% [18]. These patients are usually characterized by having many risk factors, reduced active and passive activities, and poor compliance to functional exercise, which increases their risk of developing VTE.

Therefore, it is recommended that APE should be approached as soon as possible after the operation [19]. There are still restrictions in the clinical use of APE, despite the fact that it is a simple procedure, because of individual differences, pain considerations, lack of effective APE monitoring, and lack of effective supervision.

The Ankle Pump Exercise (APE) counter system is a motion monitoring system developed by our department (Patent No. ZL 202021292479.8). It can help monitor daily ankle pump motion and carry out timed ankle pump motion reminders, since it can synchronize the movement information to the healthcare personnel, with a certain practicability. Accordingly, this study aims to explore the postoperative application of the Ankle Pump Exercise (APE) counter system in patients with femoral neck fracture.

## Study esign and methods

We selected high-and medium-risk patients with femoral neck fracture-related VTE that were treated in the Department of Orthopaedics, a Tertiary Grade A Hospital in Zhejiang province from June 2021 to June 2022. Patients were included if they (i) had lower limb fractures with an intermediate risk of developing postoperative VTE according to the Caprini assessment form, (ii) were unable to get off bed early after the operation, (iii) had a better general and mental condition postoperatively, (iv) could comply with treatment and follow-up after operation, (v) and signed the informed consent form. On the other hand, patients were excluded if they: (i) were unable to complete APE due to and underlying medical condition, (ii) did not have a definite diagnosis of VTE, (iii) had motor system abnormalities, (iv) had severe heart, lung, liver, and kidney diseases, (v) suffered from speech and communication disorders, Alzheimer’s disease, cognitive impairment or had a history of mental illnesses, and (vi) refused to participate in this study.

### Treatment and intervention

Patients in both groups were treated with routine therapy combined with postoperative ankle pump exercise. The specific steps are as follows: (i) Thromboprophylaxis: physical prevention of VTE followed by doctor’s advice; intermittent lower limb pneumatic pump combined with APE; patients were given Low Molecular Weight Heparin Sodium Injection for drug prevention according to the doctor’s advice (Qilu Pharmaceutical Factory, CFDA Approval No. H20030429, Specification: 0.4 ml:5000 iu), 2500 ~ 5000 IU hypodermic injection, once a day. (ii) Medication for pain relief: patients were given analgesics, like intravenous KETOROLAC tromethamine injection (Chengdu Bite Pharmaceutical Co., Ltd., CFDA Approval No. H20193141, Specification: 1 ml:15 mg) 15 ~ 30 mg, twice a day. (iii) Functional exercise: on the day of surgery, APE was started after waking out from anesthesia. On the first day after the operation, patients were assisted to sit up beside the bed, and use a walking aid to get out of bed and walk according to the their conditions.

### APE guidance

**Fig. 1:**
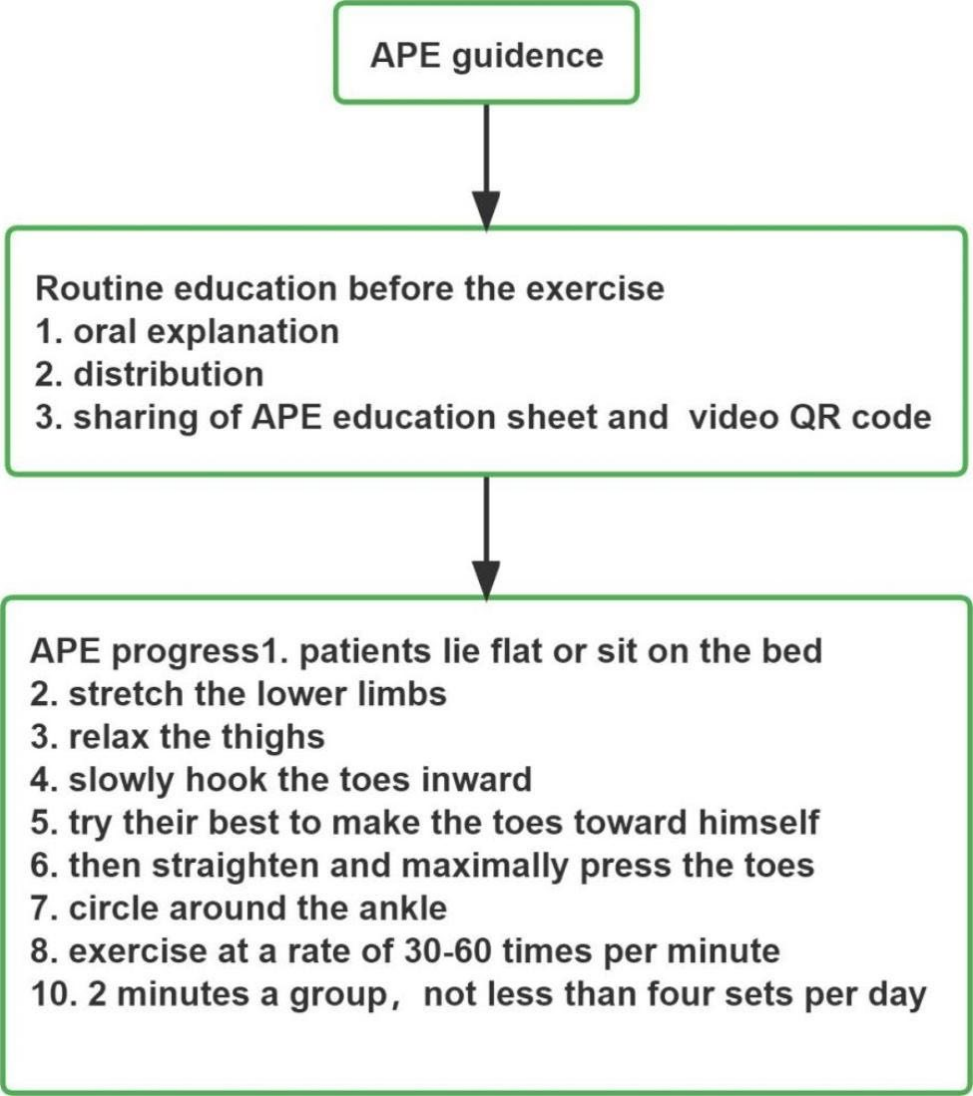
APE progress

Routine education was conducted by the nurses on the 1st postoperative day, which included oral explanation, distribution, and sharing of APE education sheet and video QR code. According the Hospital internal VTE standardized management work manual of Zhejiang nursing quality control center to formulate specific APE methods, (i) patients lie flat or sit on the bed, stretch the lower limbs, relax the thighs, slowly hook the toes inward, try their best to make the toes toward himself, and then straighten and maximally press the toes, (ii) circle around the ankle. (iii) exercise at a rate of 30–60 times per minute [20]. (iv) the exercise lasts 2 min a group. During the initial education, patients were guided by the bedside to correct the mistakes in the movement process. The frequency of exercise can be adjusted according to the patient’s physical condition, but not less than four sets per day (Fig. 1).

### Application of the ankle pump counter system

**Fig. 2:**
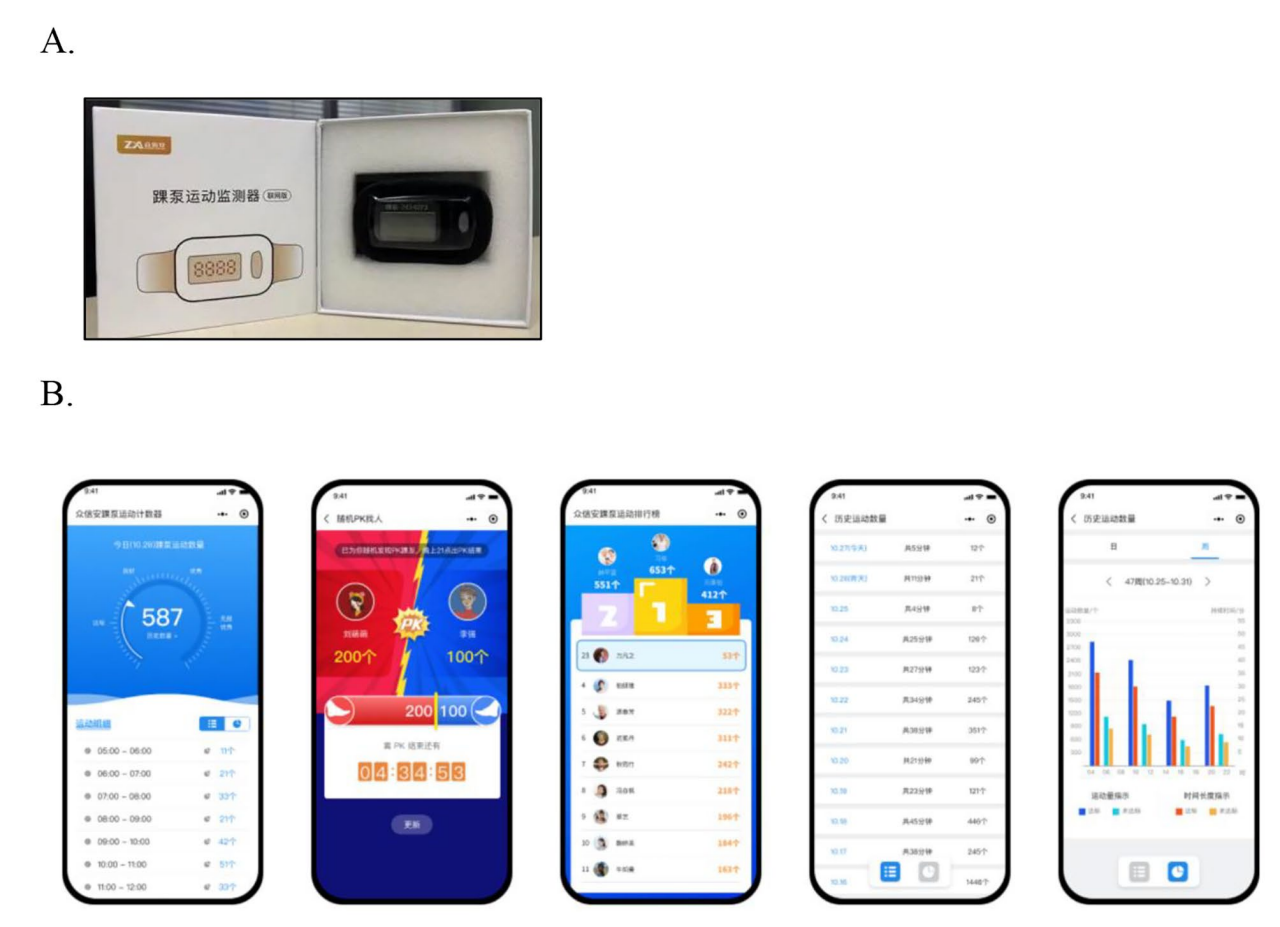
APE counter system A, APE device. B, APE counter system’s operation interface, counting, quantitative statistics

All patients in the observation group were assisted with the ankle pump counter system (patent No. ZL 202021292479.8) to perform functional exercise of ankle pump. The APE counter is used as a tool to aid in the training of regular ankle pump movements. Before the patient is able to control the WeChat program independently and follow the rhythm of the system background music for exercise, we help him bind the program by explaining how to utilize the counter. The patient terminal system will pop up a motion reminder message at regular intervals, and the system will automatically push the information four times a day to urge the patient to perform ankle pump movement. This system makes quantitative statistics on ankle pump movement, and patients can self-monitor the current movement data. The medical terminal can receive the exercise data of all patients using the counter in time and set the daily exercise goal as four daily sets. For patients with ankle pump movement < four sets or < 240 times, the program manually pushes the information again in the background to urge the patients to complete the exercise. Moreover, at 3:00 p.m. every day, we checked the patients’ movement data in the background (Fig. 2).

### Observation indicators and judgment criteria

#### Exercise compliance

Movement specifications, total exercise volume and daily exercise times of all patients were collected, considering the patient’s health status, physical strength and other factors. We created a compliance evaluation table with the following criteria: 1 point for action specification, 0 points otherwise; 1 point for each group of athletes who complete daily exercise, 0 points otherwise, and a maximum score of 4 points. For the total amount of ankle pump movement per day, 0 points were given for those whose total amount is less than 240 times, 1 point for 240–480 times, and 2 points for 480 times or above. Compliance with the daily APE was worth 6 points, while noncompliance was given no points. The compliance rates on the postoperative 1st, 3rd, and 7th days were recorded, and the compliance rate was estimated as follows: the number of compliance cases/the total number of cases×100.

### Pain score

The numerical rating scale (NRS), ranging between 0 and 10, was used to evaluate the pain degree of 15° passive hip flexion and limb rest in the postoperative 1st, 3rd, and 7th days in the two groups. Patients were each given a pain score based on how they felt, with a higher number indicating a greater degree of pain.

### Barthel index

On the first, third, and seventh days after surgery, patients’ capacity to eat, bathe, dress, toilet, regulate defecation/urination, bed and chair transfer, going up and down stairs, and walking on flat ground were scored. The score ranged between 0 and 100 and patients’ daily life abilities increases with their given scores.

#### Lower limb fatigue

The RPE scale was used to give each patient a score according to the subjective fatigue feeling of the limb on the exercise side after the exercise [21]. The scale includes 15 points from 6 to 20. Each point represents a different characteristic of fatigue feeling, and scores are given according to their own feelings. The patient’s muscle fatigue degree was recorded on the postoperative 1st, 3rd, and 7th days.

### Swelling degree

The thigh circumference of 20 cm was measured bilaterally on the upper edge of patella on the 1st and 7th postoperative days. The increased value of the affected limb’s circumference (mm) was estimated by subtracting thigh circumference of the healthy side from the affected one.

### Self-efficacy

The general self-efficacy scale (GSES) was used on the 1st preoperative and the 7th postoperative days [22]. It is comprised of ten items, and the answers were given according to the patient’s actual situation. Cronbach’s alpha indicated a level of internal consistency of 0.87 for this 10–40 point Likert scale, where a score of 1 refers totally incorrect, 2 indicates accuracy, 3 indicates high accuracy, and 4 indicates complete accuracy. On-site filling and recovery, for patients with poor education levels who are unable to fill in on their own, family members or investigators can fill in according to the patients’ requests. A total 140 questionnaires were distributed, with 100% response rate.

### Incidence of lower limb DVT

B-ultrasound was used to assess DVT incidence of both lower limbs’ veins and arteries in the two groups, and it was compared on the 7th postoperative day.

### Data analysis

Data analysis was done by SPSS 25.0. Descriptive statistics such as number, percentage, mean and standard deviation (sd) were used to describe the demographic characteristics of the participants. The Mann Whitney U test was used for non-normally distributed quantities, and the Wilcoxon signed rank test was used for inter-group comparison. *P* < 0.05 was statistically significant.

## Results

**Fig. 3:**
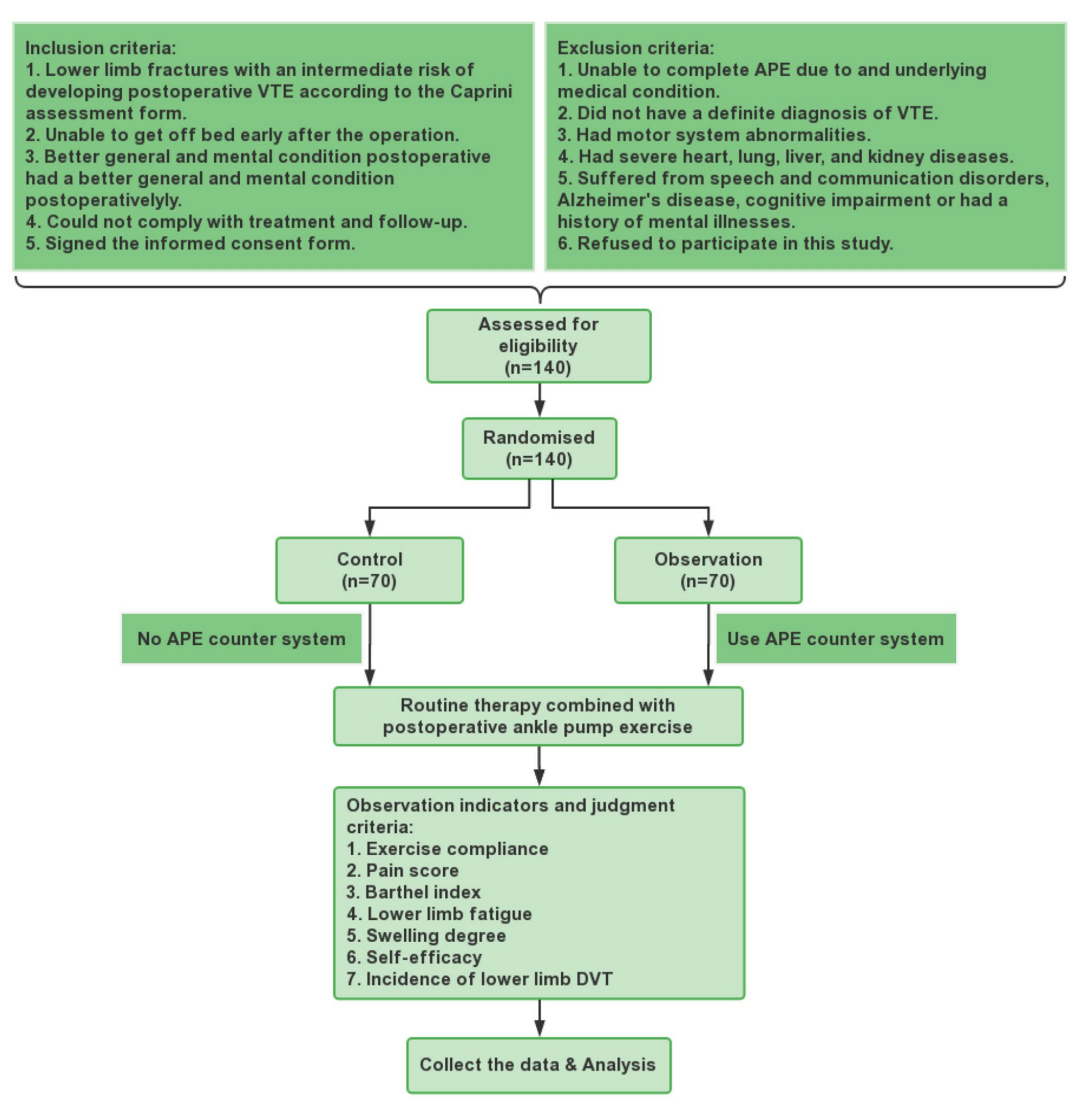
Study design

Patients were randomly divided into the control and observation groups, which included 70 cases both (study design see Fig. 3). Among the 70 cases in the observation group, 33 were males and 37 were females, with an average age of 74.17 (± 10.266) years old, values are expressed in mean ± standard. Moreover, the group included 34 cases with less than junior high school education and 36 cases with above junior high school education. Regarding operation type, 43 cases had closed reduction and internal fixation while 27 cases had hip replacement. Among the 70 cases in the control group, 31 were males and 39 were females, with an average age of 73.11 (± 10.306) years old. Furthermore, 37 cases had less than junior high school education and 33 cases with above junior high school education. Regrading operation type, 47 cases had closed reduction and internal fixation while 23 cases had hip replacement. No significant differences were noted between the two groups in age, sex, disease, education, and treatment (*P* > 0.05) (Table 1).

**Table 1:** Characteristic of participants and case control status

Variable	Overall sample	Control (n = 70)	Observation (n = 70)
Gender, n (%)			
Male	64	33 (47.14)	31 (44.29)
Female	76	37 (52.86)	39 (55.71)
χ²		0.015	
* P*		0.734	
Age(years), mean ± sd		74.17 ± 10.266	73.11 ± 10.306
t		0.608	
* P*		0.688	
Operation, n (%)			
Closed internal fixation	90	43 (61.43)	47 (67.14)
Hip replacement	50	27 (38.57)	23 (32.86)
χ²		0.498	
* P*		0.480	
Educational level, n (%)			
Junior high school and above	69	36 (51.43)	33 (47.14)
Below junior high school	71	34 (48.57)	37 (52.86)
χ²		0.257	
* P*		0.612	

There is no significant differences between the two groups in age, sex, disease, education, and treatment (*P* < 0.05)

### APE compliance contrast

Patients in the observation group had a significantly higher compliance rate than those in the control group on the 3rd and 7th days (*P* < 0.05). The compliance rate of the observation group was also higher, but non-significant, on the 1st postoperative day (Table 2).

**Table 2:** Comparison of ankle pump exercise compliance rates after operation [n (%)]

Group	1 d	3 d	7 d
Control (n = 70)	52 (74.3%)	47 (67.1%)	43 (61.4%)
Observation (n = 70)	60 (85.7%)	60 (85.7%)	58 (82.9%)
χ²	2.875	6.701	7.997
*P*	0.091	0.010	0.005

The compliance rates of the observation group was much higher than the control group on the 3rd and 7th days (*P* < 0.05)

### Pain score between the two groups

Pain quiescent condition and 15° of hip flexion did not significantly differ between the two groups on the 1st postoperative day (*P* > 0.05). After applying continuous postoperative APE, the NRS scores of patients at rest and at 15° of the hip flexion and extension were lower in the observation than the control group on the 3rd and 7th postoperative days (*P* < 0.05) (Table 3). And we also compared the amount of change of the two groups between the 7th and the 1st postoperative days, no significant difference.

**Table 3:** Pain scores of different time periods (mean ± sd)

Group	Quiescent condition			15° of flexion of the hip		
	1 d	3 d	7d	1d	3 d	7 d
Control (n = 70)	2.93 ± 0.87	2.60 ± 0.92	2.03 ± 0.92	4.34 ± 1.17	4.17 ± 1.30	3.24 ± 1.08
Observation (n = 70)	2.71 ± 0.84	2.26 ± 1.03	1.73 ± 0.99	3.96 ± 1.27	3.64 ± 1.18	2.84 ± 0.99
z	-1.671	-2.122	-2.001	-1.889	-2.803	-2.073
*P*	0.095	0.034	0.045	0.059	0.005	0.038
*Control (n = 70)	-0.90 ± 1.34			-1.10 ± 1.54		
*Observation (n = 70)	-0.99 ± 1.29			-1.11 ± 1.71		
*z	-0.487			-1.115		
*P	0.626			0.908		

The NRS scores suggested that patients at rest and at 15°of the hip flexion and extension were lower in the observation than the control group on the 3rd and 7th postoperative days (*P* < 0.05), values are expressed in mean ± sd. * represents the amount of change about pain scores between the seventh day and the first day of the two groups, no significant diffrence (*P* > 0.05)

### Barthel Index score contrast

The self-care ability scores did not significantly differ between the two groups on the 1st postoperative day (*P* > 0.05). However, the scores were significantly higher on the 3rd and 7th postoperative days were significantly higher than on the 1st postoperative day. Moreover, the scores were higher in the observation than in the control group (*P* < 0.05) (Table 4). We also compared the changes between the two groups on 1st and 7th day, and we can see observation group is significant high than the control group.

**Table 4:** Daily living self-care ability scores after operation (mean ± sd)

Group	1 d	3 d	7 d
Control (n = 70)	34.93 ± 6.40	55.29 ± 10.83	64.21 ± 8.32
Observation (n = 70)	34.43 ± 6.29	59.14 ± 10.83	67.43 ± 6.85
z	-0.627	-2.027	-3.002
*P*	0.531	0.043	0.003
*Control (n = 70)	29.29 ± 6.83		
*Observation (n = 70)	33.00 ± 11.99		
*z	-2.767		
*P	0.006		

The Barthel Index scores of the observation group was higher than the control on the 3rd and 7th days (*P* < 0.05). And both groups’ Barthel Index scores were higher than the 1st day respectively (*P* < 0.05), values are expressed in mean ± sd. * represents the amount of change about daily living self-care ability between the seventh day and the first day of the two groups (*P* < 0.05)

### Lower limb fatigue of the two groups

The lower limb fatigue scores were not significantly different between the two groups after the 1st day of exercise (*P* > 0.05). The RPE scores on the 3rd and 7th postoperative days were lower than that on the 1st day, and the scores were significantly lower in the observation than in the control group (*P* < 0.05) (Table 5). And the change of the 1st and 7th day between the two groups is meaningful.

**Table 5:** RPE scores between the two groups

Group	1 d	3d	7 d
Control (n = 70)	16.07 ± 1.48	14.44 ± 1.24	13.99 ± 1.32
Observation (n = 70)	16.21 ± 1.53	14.09 ± 1.09	13.00 ± 1.35
z	-0.664	-2.041	-4.197
*P*	0.507	0.041	0.000
*Control (n = 70)	2.09 ± 2.73		
*Observation (n = 70)	-3.21 ± 2.82		
*z	-2.419		
*P	0.016		

EPR scores indicate that the lower limb fatigue in the observation group were lower than the control group on the 3rd and 7th days (*P* < 0.05), and both lower than the 1st day respectively (*P* < 0.05), values are expressed in mean ± sd. * represents the change of the 1st and 7th day between the two groups

### Swelling degree contrast

The affected limb’s circumference was significantly reduced in the two groups on the 7th postoperative day (*P* < 0.05), and it was significantly lower in the observation than the control group (*P* < 0.05) (Table 6). Difference between the 7th and1st day of the observation group is significantly smaller than the control group.

**Table 6:** The circumference between two groups (mm, mean ± sd)

Group	1d	7 d
Control (n = 70)	56.49 ± 16.20	29.93 ± 10.07
Observation (n = 70)	56.81 ± 14.84	25.79 ± 7.88
z	-0.119	-2.068
*P*	0.905	0.039
*Control (n = 70)	-26.56 ± 10.66	
*Observation (n = 70)	-31.03 ± 11.00	
*z	2.442	
*P	0.016	

These data suggest that the swelling degree of the limbs was significantly reduced on the 7th day in both groups (*P* < 0.05), and the observation group was lower than the control on the 7th day (*P* < 0.05), values are expressed in mean ± sd. * represents the change of the 1st and 7th day between the two groups

### Comparison of self-efficacy between the two groups

Self-efficacy did not significantly differ between the two groups before APE (*P* > 0.05). Moreover, the GSES score was significantly higher in the observation than the control group on the seventh day after operation (*P* < 0.05). Moreover, the scores in the observation group was also significantly higher postoperatively than preoperatively (*P* < 0.05) (Table 7). We can see the change of the 1st and 7th day in observation is significantly higher than the control group.

**Table 7:** GSES scores between two groups (mean ± sd)

Group	Before exercise	7 day after exercise
Control (n = 70)	22.67 ± 4.17	22.97 ± 3.85
Observation (n = 70)	23.2 ± 3.52	25.31 ± 4.58
z	-1.634	-3.466
*P*	0.102	0.001
*Control (n = 70)	0.30 ± 5.17	
*Observation (n = 70)	2.11 ± 5.65	
*z	-2.395	
*P	0.017	

GSES scores indicate that the Self-efficacy of the observation group was higher than the control group on the 7th day after exercise (*P* < 0.05), values are expressed in mean ± sd. * represents the change of the 1st and 7th day between the two groups

### Incidence of lower limb DVT analysed

On the 7th postoperative day, no lower limb DVT were diagnosed in the observation group, while the control group had one case only (1.42%) (*P* = 0.316).

## Discussion

VTE is usually caused by slow blood flow, high blood coagulation, and vascular endothelial cell damage [23]. APE is mainly applied to boost blood flow speed and promote blood circulation [24]. The movement of an ankle pump is not constrained by time or place, has no significant adverse effects, and is straightforward to use with low potential risks when compared to medical prevention methods like subcutaneous injection of anticoagulants and oral anticoagulants, or physical prevention methods like elastic socks or lower limb pressure therapy [25–27]. Yet, normal postoperative ankle pump movement management can be impacted by patients’ will and inadequate efficacy.

Our study showed that patients can timely control their own activity by the APE counter. Furthermore, the sports ranking list in the system can also encourage patients to exercise initiatively, leading to enhanced patients’ self-monitoring ability and promoted patients’ active and objective exercise. By using this modality, it has also been shown that medical staff synchronously receive patients’ real-time exercise data at the medical terminal, helping them conduct health education and supervision for patients with low compliance. The timing message reminder also plays a vital role in hourly supervision, which can remarkably enhance reduced patient compliance attributed to busy work nurses, decreased timely monitoring, late-stage exercise tracking, and poor patients’ autonomy [28]. Patients self-corrected and improved exercise accuracy using intraprocedural self-paced APE instruction. Background music helps patients practice APE rhythmically, which makes it easier to master the movement frequency and focus on finishing the exercises [29]. A group exercise’s duration matches the music’s to motivate patients and ensure long-lasting movements.

Our results showed that the exercise compliance, self-care ability and self-efficacy of the observation group were significantly improved after the APE assistance, while the lower limb fatigue, pain, and the swelling degree of the observation group were significantly alleviated caompared to the control group. This suggest that our APE counter system can strengthen the self-management level, and higher self-management patients are more positive about treatment and rehabilitation. We also compared the amount of change between the first day and the seventh day about the two groups, and found that the exercise compliance, self-care ability, self-efficacy, lower limb fatigue, pain, and the swelling degree of the observation group changed more faster than the control group. All suggest that the APE counter system can better prevent the occurrence of DVT after femoral neck fracture.

Our APE counter device is non-invasive, uncomplicated, and easy to use. It can minimize bed rest-related problems, promote early recovery, shorten hospital stays, and reduce costs for patients. It can minimize nurses’ repetitious propaganda and education, reminder workload, and neglect. Also it can boost patient satisfaction and doctor-patient harmony in hospitals.

## Conclusion

In summary, APE counter system application in patients with medium and high VTE after orthopedic surgery can improve the patients’ compliance to functional exercises and self-management efficacy. However, APE’s main drawback is that it cannot substitute medications or other preventive treatments for venous thrombosis. Besides, attention should be paid to the degree of control during exercise, and excessive exercise should not be used to avoid muscle fatigue. Future studies with larger sample sizes are encouraged to extend the observation follow-up time to further observe the effect.

## Data Availability

All data generated or analysed during this study are included in this published article.

## Declarations

### Additional statement

For patients those who were illiterate, family members or investigators can fill in according to the patients’ requests.

## List of abbreviations

APE: Ankle pump exercises
VTE: Venous thromboembolism
PE: Pulmonary embolism
DVT: Deep vein thrombosis
PTS: Post-thrombotic syndrome
CFDA: China Food and Drug Administration
NRS: The numerical rating scale
GSES: The general self-efficacy scale
RPE: Rated Perceived Exertion Scale
